# Prognosis and local treatment strategies of breast cancer patients with different numbers of micrometastatic lymph nodes

**DOI:** 10.1186/s12957-023-03082-x

**Published:** 2023-07-10

**Authors:** Shiping Luo, Wenfen Fu, Jingyi Lin, Jie Zhang, Chuangui Song

**Affiliations:** 1 Department of Breast Surgery, Fujian Medical University Union Hospital, Fuzhou, Fujian Province, China; 2 Breast Cancer Institute, Fujian Medical University, Fuzhou, Fujian Province, China; 3 Department of General Surgery, Fujian Medical University Union Hospital, Fuzhou, Fujian Province, China

**Keywords:** N1mi breast cancer, Lymph node micrometastases (LNMM), Sentinel lymph nodes biopsy (SLNB), Axillary lymph nodes dissection (ALND), Local radiotherapy

## Abstract

**Background:**

Lymph node micrometastasis is an important prognostic factor in breast cancer, but patients with different numbers of involved lymph nodes are all divided into the same N1mi stage without distinction. We designed this study to compare the prognosis and local treatment recommendations of N1mi breast cancer patients with different numbers of micrometastatic lymph nodes.

**Patients and methods:**

A total of 27,032 breast cancer patients with T1-2N1miM0 stage from the Surveillance, Epidemiology, and End Results (SEER) database (2004–2019) who underwent breast surgery were included in this retrospective study. Patients were divided into three groups for prognosis comparison according to the number of micrometastatic lymph nodes: N1mi with 1 (Nmi = 1), 2 (Nmi = 2), or more (Nmi ≥ 3) involved lymph nodes. We explored the characteristics and survival outcomes of the population receiving different local treatments, including different axillary surgery types and whether receiving radiotherapy or not. Univariate and multivariate Cox proportional hazards regression analysis were used to compare the overall survival (OS) and breast cancer-specific survival (BCSS) in different groups. Stratified analyses and interaction analyses were also applied to explore the predictive significance of different involved lymph nodes numbers. Propensity score matching (PSM) method was utilized to balance the differences between groups.

**Results:**

Univariate and multivariate Cox regression analysis indicated that nodal status was an independent prognostic factor. After adjustment for other prognostic factors, there was a significant difference in prognosis between Nmi = 1 group and Nmi = 2 group [adjusted hazard ratio (HR) 1.145, 95% confidence interval (CI): 1.047–1.251, *P* = 0.003], and patients with Nmi ≥ 3 group had a significantly poorer prognosis (adjusted HR 1.679, 95% CI 1.589–2.407; *P* < 0.001). The proportion of N1mi patients only underwent sentinel lymph nodes biopsy (SLNB) gradually increased from 2010 (*P*_trend_ < 0.001). After adjusting for other factors, N1mi patients who underwent axillary lymph nodes dissection (ALND) was associated with significant survival benefit than SLNB (adjusted HR 0.932, 95%CI 0.874–0.994; *P* = 0.033), the same goes for receiving radiotherapy (adjusted HR 1.107, 95%CI 1.030–1.190; *P* = 0.006). Further stratified analysis showed that in the SLNB subgroup, radiotherapy was associated with a significant survival benefit (HR 1.695, 95%CI 1.534–1.874; *P* < 0.001), whereas in the ALND subgroup, there was no significant prognostic difference with or without radiotherapy (HR 1.029, 95%CI 0.933–1.136; *P* = 0.564).

**Conclusion:**

Our study indicates that the increasing number of lymph node micrometastases was associated a worse prognosis of N1mi breast cancer patients. In addition, ALND does provide a significant survival benefit for these patients, while the benefit from local radiotherapy may be of even greater importance.

**Supplementary Information:**

The online version contains supplementary material available at 10.1186/s12957-023-03082-x.

## Introduction

Lymph node micrometastases (LNMM) was defined as the presence of metastases no larger than 2 mm in the lymph nodes, which was firstly proposed by Huvos et al. in 1971 [1]. In 2002, the 6th edition of the American Joint Committee on Cancer (AJCC) Manual for Staging of Cancer based on lymph node involvement status, classified N staging into macrometastases (metastases lager than 2 mm), micrometastases (N1mi, metastases 0.2–2 mm in size), and isolated tumor cells (ITC, single tumor cells or small clusters of cells not larger than 0.2 mm, pN0(i +)) [2, 3]. After that, Patani et al. [4, 5] analyzed relevant literature on breast cancer LNMM, found that 12 of them (2000–2006) supported LNMM with independent prognostic significance and the prognosis is worse than that of lymph nodes without metastasis. While a recent multi-center cohort study from Sweden [6] showed that lymph node micrometastases were associated with significantly lower 10-year breast cancer-specific survival (BCSS) and overall survival (OS) rates compared with lymph node-negative cases, while outcomes were similar to those of lymph node macrometastases, which may be associated with inadequate systemic treatment. Although the conclusions of different studies are inconsistent, LNMM is still considered to be an important prognostic factor of breast cancer.

With the progress of comprehensive treatments of breast cancer, surgical treatment has gradually become more precise and less invasive. Hence, the local treatments of patients with LNMM have attracted more attention to further improve. Previous large clinical studies [7–11] have attempted to address the question of whether axillary lymph node dissection (ALND) can be safely omitted when micrometastases are found in sentinel lymph nodes (SLN). Although the 10-year follow-up results of IBCSG 23–01 [8] and the American College of Surgeons Oncology Group(ACOSOG) Z0011 trial [9] both support the avoidance of ALND in breast cancer patients with LNMM, most of these patients underwent breast-conserving surgery (BCS) plus whole breast radiation therapy (WBRT). Therefore, the conclusions of these two studies can only be applied to the clinical practice of relevant populations that meet the inclusion criteria and cannot be extrapolated to all pN1mi patients. In addition, the AMAROS trial [10] showed that axillary radiotherapy (ART) is the best alternative to ALND in patients with 1–2 sentinel lymph node (SLN) metastases.

Accordingly, National Comprehensive Cancer Network(NCCN) guideline [12] and American Society of Clinical Oncology(ASCO) guideline [13] recommend radiotherapy of the axilla in lieu of ALND in patients with pathological SLN-positive and low tumor burden. Patients with LNMM are also treated in the light of the guidelines’ recommendation for patients with positive lymph nodes. However, different numbers of involved lymph nodes are all divided into the same N1mi stage without categorization. We designed this study to compare the prognosis and local treatment recommendations of N1mi breast cancer patients with different numbers of micrometastatic lymph nodes.

## Patients and methods

### Data source and study population

We screened the Surveillance, Epidemiology, and End Results (SEER) database of the National Cancer Institute to identify eligible breast cancer patients in this retrospective study. The SEER database is an open-access resource for cancer-based epidemiology and survival analyses (See Website “https://seer.cancer.gov/data/” for detailed information). Data access for present study was authorized by SEER Program. As all patient information in the SEER database is de-identified, this study was exempt from Institutional Review Board evaluation.

The SEER*Stat version 8.4.0 was utilized to extract 32,032 pN1mi breast cancer patients’ information, diagnosed between January 2004 and December 2019 (Nov 2021 Submission). We excluded patients identified by death certificate or autopsy and with incomplete survival data. Female patients with T1-2 invasive breast cancer without distant metastasis and underwent breast surgery were included in this study. Patients who did not undergo surgery or whose type of surgery was unknown, had distant metastases, or had an unclear number of axillary lymph nodes examined were excluded (Supplemental Figure S1). The data elements include patient basic demographic characteristics, cancer pathological types, staging and molecular biomarkers status, the treatment received for the cancer, and survival outcomes information. It is worth noting that the information of epidermal growth factor receptor-2 (HER2) status in the SEER database has been registered from 2010, so the correlation analysis of HER2 status and molecular types only included case data since then.

Finally, a total of 27,032 female breast cancer patients, with a stage of T1-2N1miM0, were included in our retrospective study. Patients were divided into three groups for prognosis comparison according to the number of LNMM: N1mi with 1 (Nmi = 1), 2 (Nmi = 2), or more (Nmi ≥ 3) involved lymph nodes. Furthermore, survival outcomes of different local treatments, including axillary surgery types and radiotherapy, are required for these three groups. The number of lymph nodes removed was used as a surrogate for the type of axillary surgery which was defined as in previous similar studies [14–17], that is, patients with 5 or less lymph nodes resected were categorized as receiving sentinel lymph node biopsy (SLNB) while 6 or more as undergoing ALND.

### Statistical analysis

Patients-, tumor-, and treatment- level characteristics are presented as frequencies (N) and percentages, and compared using chi-square tests as appropriate. Kaplan–Meier method and log-rank test were used to draw survival curves and compare differences among different subgroups without adjustment for other factors. Univariate and multivariate cox regression analyses were used to identify independent prognostic factors and to calculate hazard ratios (HR) toward target subgroups after adjustment for other prognostic factors. Stratified analyses and interaction analyses were also applied to explore the predictive significance of different LNMM involved numbers.

In the analysis of axillary surgery and radiotherapy, we adopted the method of 1:1 nearest propensity score matching (PSM) with matching tolerance 0.02, in order to balance the characteristic differences between the two compared axillary surgery groups, covariables included in propensity score matching were age, race, marital status, grade, T stage, nodal status, estrogen receptor (ER) status, progesterone receptor (PR) status, HER2 status, type of breast surgery, radiation and chemotherapy. All tests were two-sided, and a *P* value < 0.05 was considered to be statistically significant. All statistical analyses were performed using IBM SPSS software version 24.0 (IBM Corp., Armonk, USA) and R version 4.1.3 (The R Project for Statistical Computing, Vienna, Austria).

## Results

### Basic characteristics and survival analyses of the overall population

A total of 27,032 patients with T1-2N1miM0 breast cancer were included in this study, of which 22,463 (83.1%) were involved in one LNMM, 3,089 (11.4%) in two, and 1,480 (5.5%) in three or more lymph nodes. The patient’s basic characteristics are shown in Table 1. There were 21,466 (77.9%) patients with pathological type of invasive ductal carcinoma (IDC) and 2123 (7.9%) of invasive lobular carcinoma (ILC). Among all the patients, 59.1% had tumors no larger than 2 cm in size, and the remaining 40.9% had tumors between 2 and 5 cm. Most patients were hormone receptor positive (ER positive 85.9%, progesterone receptor (PR) positive 76.3%) and HER2 negative (84.1%, after 2010). A total of 15,622(57.8%) patients underwent SLNB, of which 14,293(91.5%) were patients with one LNMM, accounting for 63.6% of the Nmi = 1 subgroup. While in the Nmi = 2 subgroup, the proportion of receiving SLNB decreased to 38.1%, and the remaining 61.9% of patients received ALND.

**Table 1:** Demographic and clinical characteristics of patients, stratified by the number of micrometastatic lymph nodes

Characteristics	Total	N1mi = 1	N1mi = 2	N1mi ≥ 3	*P* value
*N*	27,032	22,463	3089	1480	
Age, years					< 0.001
< 40	1838 (6.8)	1463 (6.5)	250 (8.1)	125 (8.4)	
40–59	13,141 (48.6)	10,813 (48.1)	1567 (50.7)	761 (51.4)	
≥ 60	12,053 (44.6)	10,187 (45.4)	1272 (41.2)	594 (40.1)	
Race					< 0.001
White	21,466 (79.4)	17,954 (79.9)	2398 (77.6)	1114 (75.3)	
Black	2793 (10.3)	2198 (9.8)	379 (12.3)	216 (14.6)	
Other ^a^	2773 (10.3)	2311 (10.3)	312 (10.1)	150 (10.1)	
Marital					0.003
Married	16,130 (59.7)	13,501 (60.1)	1793 (58.0)	836 (56.5)	
Single ^b^	9822 (36.3)	8094 (36.0)	1147 (37.1)	581 (39.3)	
Unknown	1080 (4.0)	868 (3.9)	149 (4.8)	63 (4.3)	
Histological types					0.038
IDC	21,070 (77.9)	17,565 (78.2)	2377 (77.0)	1128 (76.2)	
ILC	2123 (7.9)	1725 (7.7)	254 (8.2)	144 (9.7)	
Other	3839 (14.2)	3173 (14.1)	458 (14.8)	208 (14.1)	
Grade					< 0.001
I	5252 (19.4)	4596 (20.5)	496 (16.1)	160 (10.8)	
II	12,950 (47.9)	10,786 (48.0)	1483 (48.0)	681 (46.0)	
III	8167 (30.2)	6578 (29.3)	1013 (32.8)	576 (38.9)	
Unknown	663 (2.5)	503 (2.2)	97 (3.1)	63 (4.3)	
T stage					< 0.001
T1	15,986 (59.1)	13,758 (61.2)	1613 (52.2)	615 (41.6)	
T2	11,046 (40.9)	8705 (38.8)	1476 (47.8)	865 (58.4)	
Type of surgery					< 0.001
BCS	14,826 (54.8)	12,768 (56.8)	1522 (49.3)	536 (36.2)	
Mastectomy	12,206 (45.2)	9695 (43.2)	1567 (50.7)	944 (63.8)	
Type of axillary surgery					< 0.001
SLNB	15,622 (57.8)	14,293 (63.6)	1176 (38.1)	153 (10.3)	
ALND	11,410 (42.2)	8170 (36.4)	1913 (61.9)	1327 (89.7)	
Radiation					0.181
Yes	14,368 (53.2)	11,995 (53.4)	1598 (51.7)	775 (52.4)	
No/refused	12,664 (46.8)	10,468 (46.6)	1491 (48.3)	705 (47.6)	
Chemotherapy					< 0.001
Yes	14,039 (51.9)	11,021 (49.1)	1913 (61.9)	1105 (74.7)	
No/unknown	12,993 (48.1)	11,442 (50.9)	1176 (38.1)	375 (25.3)	
ER status					< 0.001
Positive	23,208 (85.9)	19,446 (86.6)	2593 (83.9)	1169 (79.0)	
Negative	3271 (12.1)	2586 (11.5)	421 (13.6)	264 (17.8)	
Borderline	553 (2.0)	431 (1.9)	75 (2.4)	47 (3.2)	
PR status					< 0.001
Positive	20,631 (76.3)	17,285 (76.9)	2315 (74.9)	1031 (69.7)	
Negative	5649 (20.9)	4584 (20.4)	675 (21.9)	390 (26.4)	
Borderline	752 (2.8)	594 (2.6)	99 (3.2)	59 (4.0)	
HER2 status					< 0.001
Positive	2048 (7.6)	1690 (7.5)	243 (7.9)	115 (7.8)	
Negative	13,957 (51.6)	12,044 (53.6)	1409 (45.6)	504 (34.1)	
Borderline	600 (2.2)	488 (2.2)	64 (2.1)	48 (3.2)	
Not 2010 +	10,427 (38.6)	8241 (36.7)	1373 (44.4)	813 (54.9)	

*IDC* invasive ductal carcinoma, *ILC* invasive lobular carcinoma, *SLNB* sentinel lymph node biopsy, *ALND* axillary lymph node dissection, *BCS* breast-conserving surgery, *ER* estrogen receptor, *PR* progesterone receptor; *HER2* human epidermal growth factor receptor 2

^a^Other includes American Indian/Alaskan native, and Asian/Pacific Islander

^b^Single includes divorced, separated, single (never married), unmarried or domestic partner and widowed

**Fig. 1:**
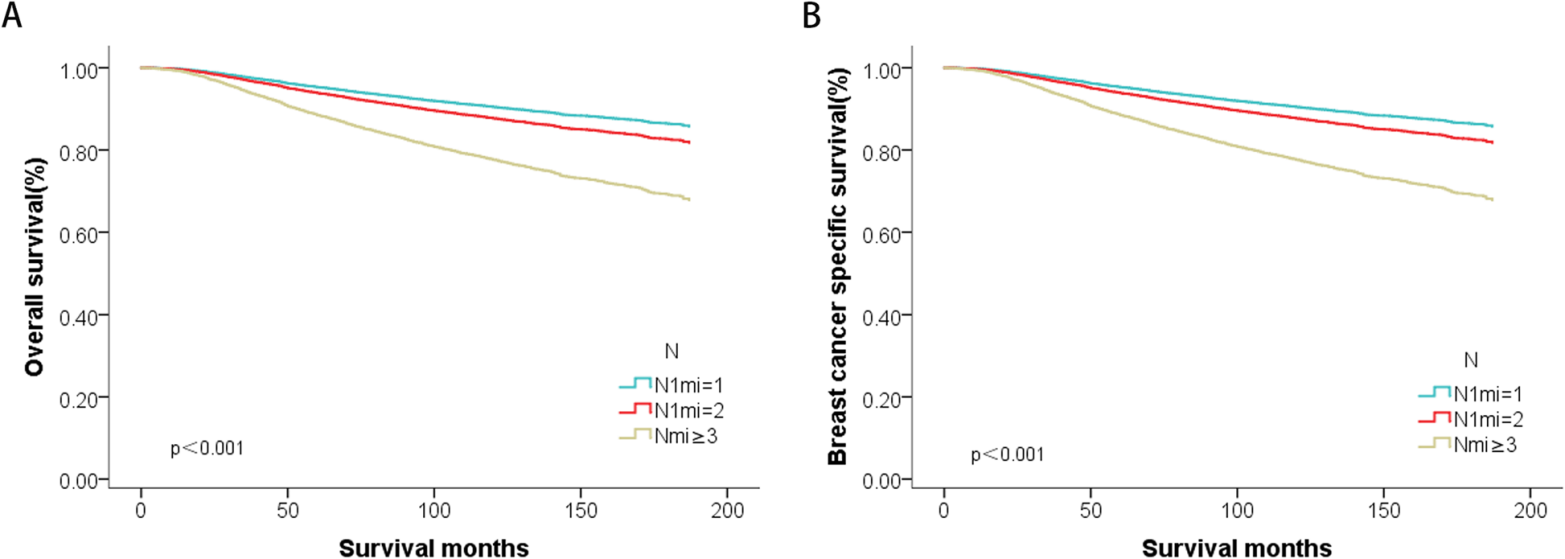
The survival curves of adjusted by other prognostic factors (**A**: overall survival; **B**: breast cancer specific survival), stratified by different numbers of involved lymph nodes

The univariate and multivariate cox regression analysis (Supplemental Table S1) indicated that age, race, marital status, histologic type, grade, T stage, number of LNMM, ER, PR, HER2 status, and different type of adjuvant treatments were independent prognostic factors in the T1-2N1miM0 breast cancer population. After adjustment for other prognostic factors, the overall death risk of Nmi = 2 increased by 1.145 times (95%CI 1.047–1.251, *P* = 0.003) compared with Nmi = 1, and the risk of Nmi ≥ 3 group increased by 1.697 times (95%CI 1.53–1.882, *P* < 0.001) (Fig. 1). Therefore, within the same pN1mi stage, the prognosis of different numbers of LNMM was significantly different.

### Descriptive statistics and survival analyses of axillary surgery types

**Fig. 2:**
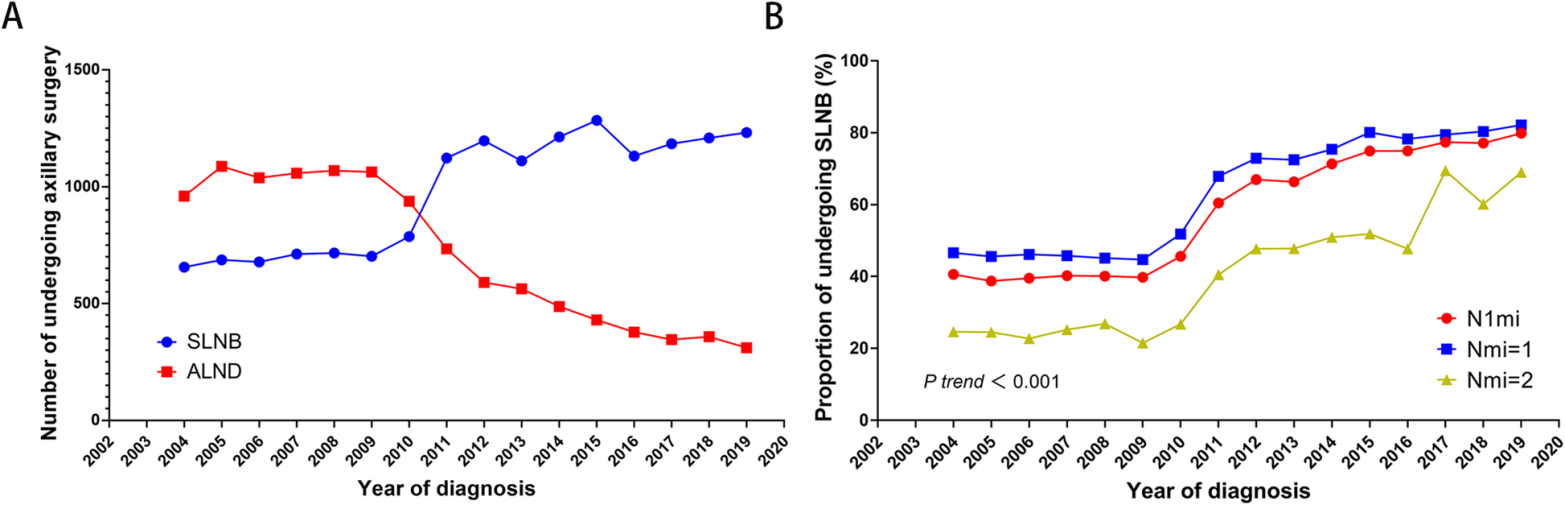
Tendency of patients with T1-2N1miM0 breast cancer undergoing different types of axillary surgery between 2004 and 2019. **A** The number of undergoing SLNB and ALND. **B** The proportion of undergoing SLNB

Baseline characteristics for different types of axillary surgery are presented in Supplemental Table S2. It is shown that in the T1-2N1miM0 population, patients older than 60 were more likely to receive SLNB, and patients younger than 40 were more likely to undergo ALND. In addition, patients underwent breast-conserving surgery (BCS) were more likely to receive SLNB, while ALND was more common in patients with mastectomy. In Fig. 2, it can be clearly seen that before 2010, most of the patients with pN1mi received ALND. And after 2011, the number of patients has gradually decreased. The number of different axillary surgery types reversed between 2010 and 2011. Overall, the proportion of pN1mi patients receiving SLNB is increasing year by year(*P* for trend < 0.001). Among them, the patients with one LNMM are the most significant (Supplemental Table S3).

**Fig. 3:**
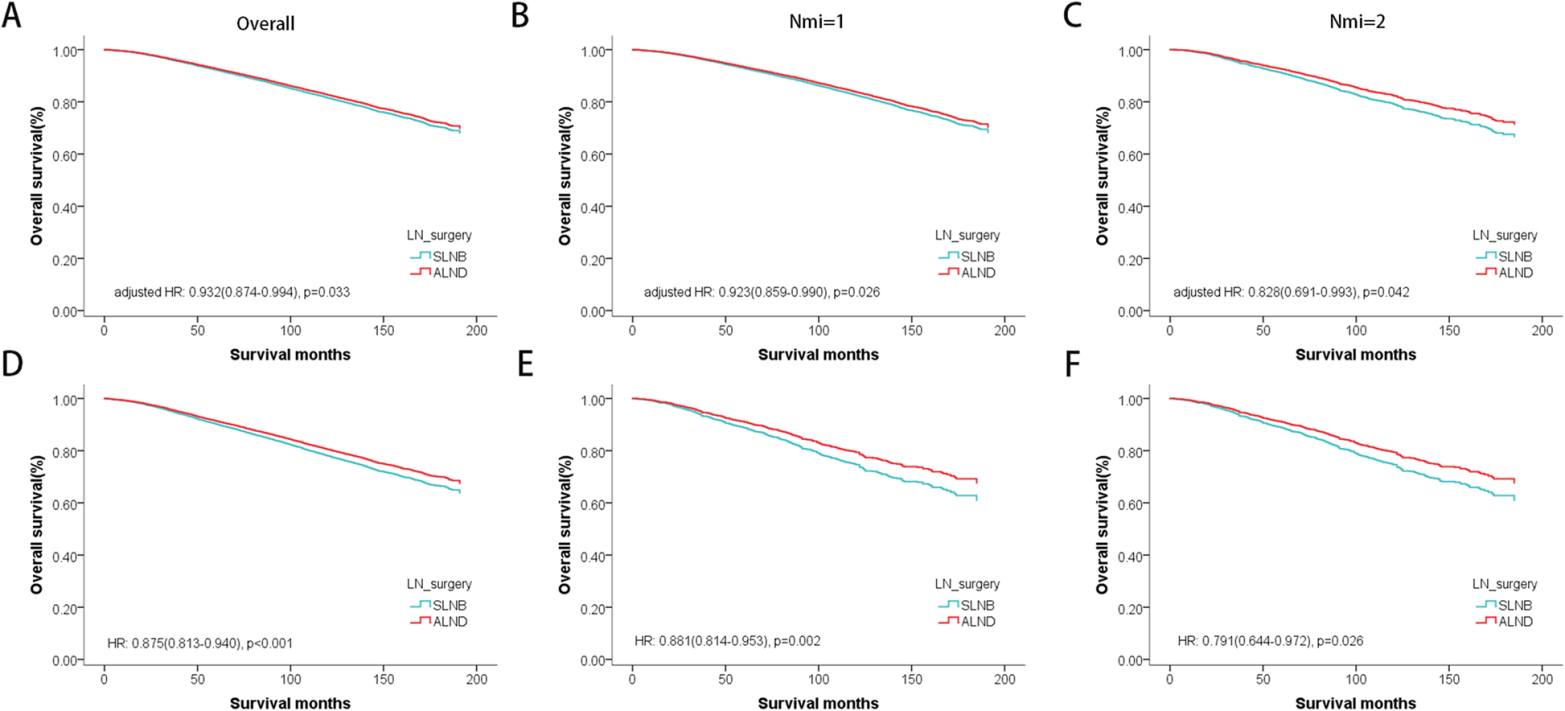
The survival curves of different axillary surgery types. **A**–**C** Adjusted by other prognostic factors. **D**–**F** After propensity score matching

However, in the overall population, ALND was associated with better overall survival than SLNB (adjusted HR 0.932, 95%CI 0.874–0.994; *P* = 0.033) (Supplemental Table S1), this trend was also the same in the Nmi = 1 subgroup (adjusted HR: 0.926, 95%CI 0.859–0.990; *P* = 0.026) and the Nmi = 2 subgroup(adjusted HR 0.828, 95%CI 0.691–0.993; *P* = 0.042) (Fig. 3A–C). To further verify this conclusion, we performed 1:1 PSM on SLNB and ALND cohorts, and there was ditto significant difference in survival between the two groups after matching (HR 0.875, 95%CI 0.813–0.940; *P* < 0.001). The same result was found in the Nmi = 1 subgroup (HR 0.881, 95%CI 0.814–0.953; *P* = 0.002), and the Nmi = 2 subgroup (HR 0.791, 95%CI 0.644–0.972; *P* = 0.026) (Fig. 3D–F).

### Stratified analyses of radiation and interaction analyses

**Fig. 4:**
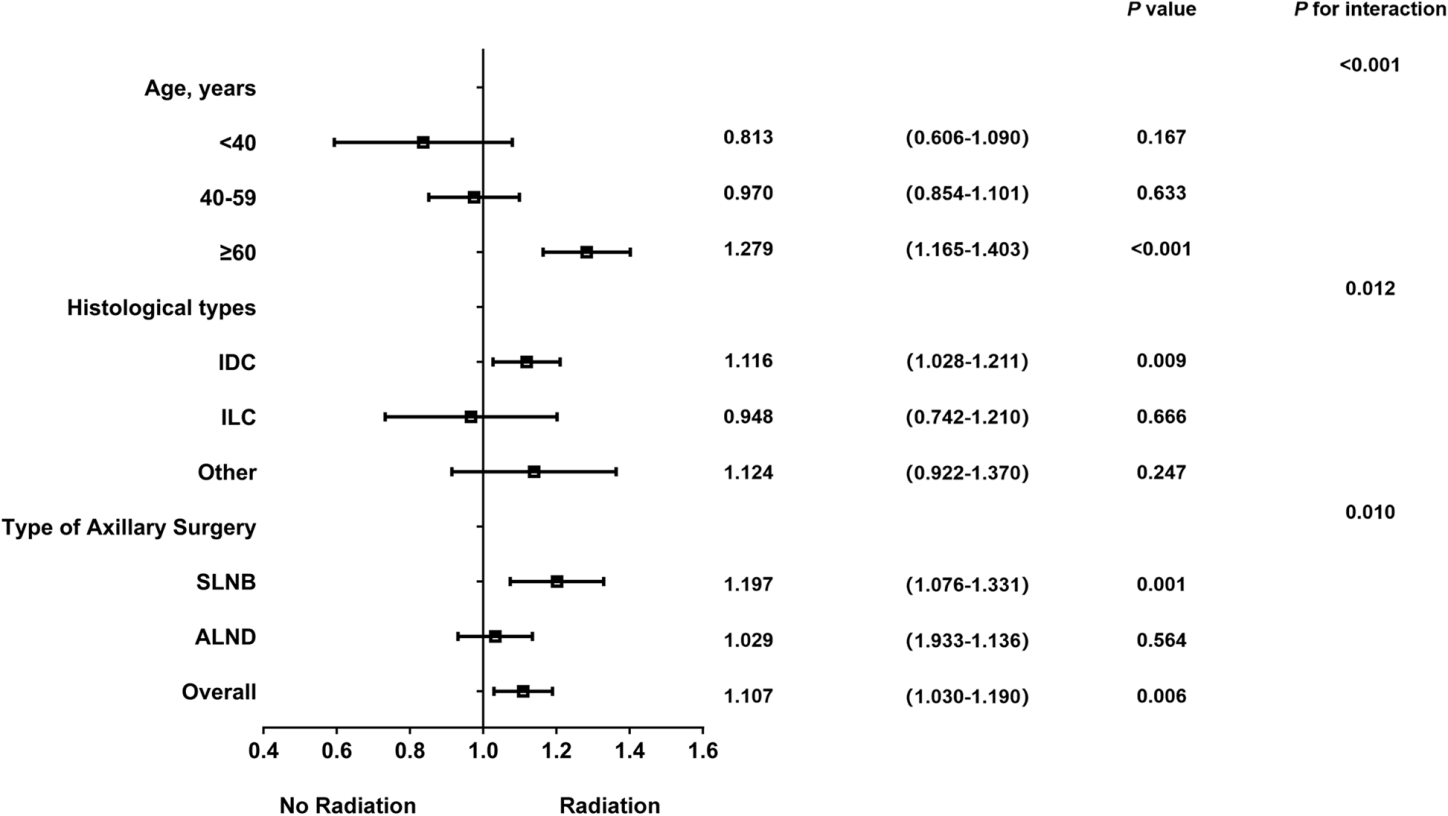
Stratified analyses and interaction analyses of receiving local radiotherapy

After adjustment for other factors, receiving radiotherapy resulted in some improvement in prognosis (HR 1.107, 95%CI 1.030–1.190; *P* = 0.006) (Fig. 4). Further stratification analyses showed that the benefit of radiation was significant for IDC patients (HR 1.116, 95%CI 1.028–1.211; *P* = 0.009), but not for ILC (HR: 0.948, 95%CI 0.742–1.210; *P* = 0.666) and other types of carcinoma. In the SLNB subgroup, radiotherapy was associated with a significant survival benefit (adjusted HR 1.197, 95%CI 1.076–1.331, *P* = 0.001), whereas in the ALND subgroup, there was no significant prognostic difference with or without radiotherapy (HR 1.029, 95%CI 0.933–1.136; *P* = 0.564) (Supplemental Figure S2).

**Fig. 5:**
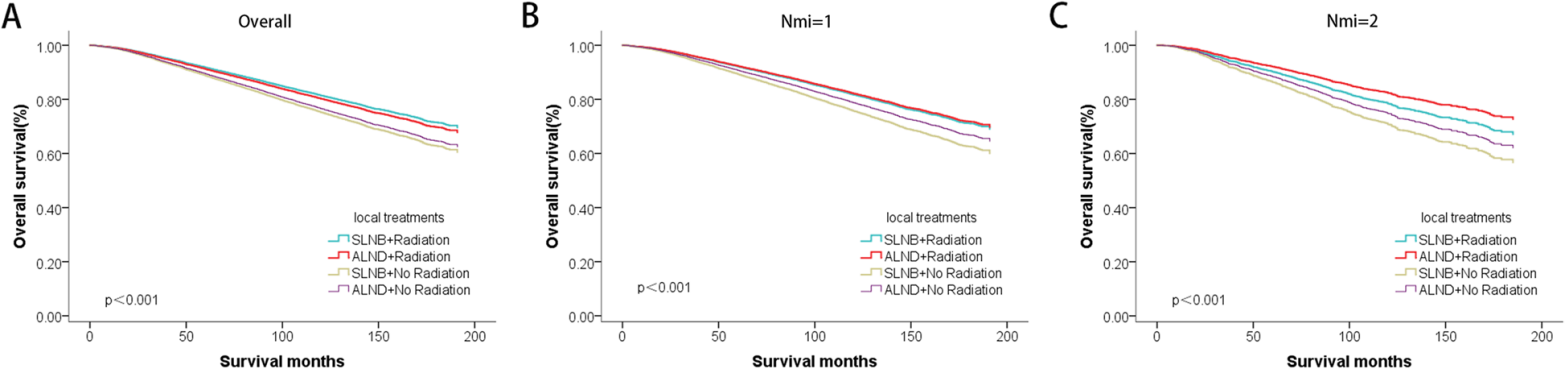
The survival curves of different local treatments combinations, including axillary surgery types and local radiotherapy

We performed an interaction analysis between axillary surgery type with radiotherapy and obtained significant results. This means that the radiotherapy benefit difference between the with and without radiotherapy in the SLNB and ALND subgroups is distinct. According to the survival analysis (Fig. 5) of different local treatments combinations, it appears that receiving SLNB plus radiation has the best prognosis, while SLNB without radiotherapy gain the worst prognosis. There was no significant difference in survival outcomes between SLNB and ALND with radiotherapy (*P* = 0.121), nor between the two groups without radiotherapy (*P* = 0.113). However, the difference in survival between radiotherapy and no radiotherapy was prominent. When the number of LNMM was only one, the conclusion was consistent with the above. However, when two lymph nodes were involved, the combination of ALND plus radiotherapy transformed the best prognosis.

## Discussion

From the 6th edition of AJCC manual [2] for staging to the latest 8th edition [14], no matter how many number of micrometastasis lymph nodes were detected, they were all divided into the same pN1mi staging, and the difference in prognosis caused by the inconsistent number was not distinguished. Evidence that the number of macrometastatic lymph nodes negatively affects survival outcome [18–20] prompted refinement of the staging system. We designed this study to distinguish the prognosis and local treatment recommendations of N1mi breast cancer patients with different numbers of micrometastatic lymph nodes involved. Our study demonstrated that for breast cancer patients with identical T1-2N1miM0 stage, the greater number of LNMM, the worse the prognosis (*P* < 0.001).

To investigate the prognostic significance of axillary lymph node micrometastases, the MIRROR trial [21] was the first retrospective cohort study of patients with LNMM and ITC, which confirmed that for patients who did not receive adjuvant therapy, both pN0(i +) and pN1mi stages are independent prognostic indicators. There is no significant survival difference in prognosis between pN0(i +) and pN1mi stage patients, and both pN0(i +) and pN1mi stage patients can benefit from adjuvant therapy. The NSABP B-32 trial [11, 22] enrolled 3795 breast cancer patients who underwent BCS and received postoperative whole-breast radiotherapy(WBRT) and systemic adjuvant therapy. After a median follow-up of 95 months, there was no significant difference in disease-free survival (DFS), OS and distant metastasis-free survival between pN1mi and pN0 stage patients. There are also discussions on the prognostic significance of the involved lymph node number in patients with pNmi stage. Roi Weiser et al. [15] used National Cancer Database (NCDB) data to analyze the prognosis of lymph nodes status, and concluded all nodal status had a positive effect on survival compared with Nmic > 1 status, with HRs of 0.68, 0.88, and 0.93 for N0, Nmi = 1, and N1.1 disease respectively, with only N0 reaching statistical significance.

Through the SEER database registration data, it can be observed that before 2010, more pNmi patients chose to receive ALND, but this situation changed in 2011, and SLNB only became a preferred option for more patients, and the proportion of undergoing SLNB has since increased year by year. Until the most recent follow-up in 2019, about 79.84% of patients only received SLNB, and the proportion of patients with only one LNMM was as high as 82.14%. The management of the axilla in patients with LNMM has a long history. In 2010, Yi et al. [23] reported a retrospective study on the choice of SLNB or ALND in patients with axillary lymph node-positive breast cancer. There were 6838 breast cancer patients with LNMM, of which 2240 received SLNB and 4598 received ALND, and post-surgery relevant systemic therapy and local radiation therapy. After 50 months of follow-up, there was no significant difference in the recurrence rate between patients who underwent SLNB only and those who underwent ALND. However, in our study, ALND does provide a significant survival benefit for N1mi breast cancer patients after a median follow-up of 95 months, whether in multivariate-adjusted cox regression analyses or survival analyses after PSM.

Both IBCSG 23–01 [8] and ACOSOG Z0011 [9] suggest that axillary dissection can be avoided in patients with early breast cancer and limited sentinel lymph node involvement. However, since more than 90% of the patients received BCS + whole breast radiotherapy in these two trials, the results are only applicable to the status of the enrolled population. AATRM trial [24] is a prospective and randomized clinical trial specifically targeting the early breast cancer patients with sentinel lymph node micrometastases, it randomized patients to ALND or clinical follow-up and showed no significant difference in DFS between two groups. Another multi-institutional prospective study of 260 pT1-2Nmi post-mastectomy patients reported from Lim SZ et al. [25] suggested that no statistically significant differences were found between patients with SLNB, ALND, or PMRT. Nonetheless, our study found discrepant results with different numbers of LNMM involved. SLNB with radiotherapy had the best prognosis when there was only one lymph node micrometastasis, while when the number of micrometastatic lymph nodes increased to two, ALND plus radiotherapy had the best survival outcome. Although only whether received radiotherapy or not achieved significant benefit, it still suggested that when the number of lymph node involved is different, the treatment mode should be focused and cannot be static.

The recently published prospective SENOMIC trial [26] omitting a completion ALND in breast cancer patients with sentinel LNMM, and found that patients who had mastectomy without adjuvant radiotherapy had a significantly higher risk of recurrence than those who underwent breast-conserving surgery. As with trials such as IBCSG23-01 and Z0011, radiotherapy after BCS plays an important role in improving outcomes. In our stratification analysis of different treatments, we found that there was no significant difference in survival between different axillary surgery types, with or without radiotherapy. While when patients have undergone different types of axillary surgery, whether they receive radiotherapy become important. When pNmi breast cancer patients only underwent SLNB, compared with those who received radiotherapy, the HR value of without radiotherapy was 1.695 (95%CI 1.534–1.874; *P* < 0.001).

Stratified analyses and interaction analysis in our study indicate that the benefit from local radiotherapy in pN1mi patients may be of even greater importance on the survival outcome. AMAROS trial [10] testified axillary radiotherapy is the best option to replace ALND when 1–2 SLNs have metastasized in T1-2 breast cancer patients, which can improve the quality of life without affecting DFS and OS. The OTOASOR trial [27] also have proved the equivalence of ALND and ART in patients with low lymph nodal burden. These two trials included 29% and 25% of patients with microscopic nodal disease respectively. In 2018, Wu SP et al. [28] reported a retrospective study that evaluated the survival impact of PMRT in patients with N1mi within the National Cancer Database, and found that no OS differences were associated with PMRT, whether in the SLNB group or the ALND group. Another two large, single-institution studies separately from Memorial Sloan Kettering Cancer Center and MD Anderson Cancer Center [29, 30] demonstrated no difference in local recurrence rates(LRR) for patients with N1mi disease post-mastectomy and SLNB, regardless of further radiation or ALND. However, a study from Merfeld EC et al. [31] indicated that pN1mi patients with grade 3 were observed to be at substantial risk for LRR, and radiotherapy was associated with a lower risk of LRR.

Inevitably, there are several limitations related to its design and data source in our study. Firstly, this is a retrospective study derived from a public database, although PSM-based analyses can reduce the effects of the observed confounders, it cannot address unobserved confounders nor the inevitable cases-loses. Secondly, locoregional recurrence cannot be captured in the SEER database. And it is also unfortunate that cases receiving neoadjuvant chemotherapy (NAC) could not be identified in the SEER database, so axillary management cannot be discussed in patients receiving NAC.

## Conclusion

Our study indicates that the increasing number of LNMM was associated a worse prognosis of N1mi breast cancer patients. And ALND does provide a significant survival benefit for N1mi patients, while the benefit from local radiotherapy may be of even greater importance, avoidance of ALND can be considered in patients receiving radiotherapy. In addition, local treatment strategies for breast cancer patients with different numbers of micrometastatic lymph nodes should be individualized and cannot be generalized.

## Supplementary Information

**Additional file 1: Supplemental Figure S1. **Flow diagram of identifying eligible patients with T1-2N1miM0 breast cancer.

**Additional file 2: Supplemental Figure S2. **The survival curves of receiving radiotherapy or not, adjusted by other prognostic factors.

**Additional file 3: Supplemental Table S1. **Univariate and multivariate analyses of overall survival (OS) in T1-2N1miM0 patients.

**Additional file 4: Supplemental Table S2. **Baseline characteristics of before and after matching in patients, stratified by type of axillary surgery.

**Additional file 5: Supplemental Table S3. **The number of patients with T1-2N1miM0 breast cancer undergoing different types of axillary surgery between 2004 and 2019.

**Additional file 6: Supplemental Table S4. **Baseline characteristics of before and after matching in patients, stratified by receive radiation or not.

## Data Availability

Publicly available datasets were analyzed in this study. This data can be found here: Surveillance, Epidemiology, and End Results (SEER) database (https://seer.cancer.gov/).

## Abbreviations

OS: Overall survival
BCSS: Breast cancer-specific survival
PSM: Propensity score matching
LNMM: Lymph node micrometastases
SLNB: Sentinel lymph node biopsy
ALND: Axillary lymph node dissection
BCS: Breast-conserving surgery
ER: Estrogen receptor
PR: Progesterone receptor
HER2: Human epidermal growth factor receptor 2
IDC: Invasive ductal carcinoma
ILC: Invasive lobular carcinoma
WBRT: Whole breast radiation therapy
ART: Axillary radiotherapy
HR: Hazard ratio
CI: Confidence interval
DFS: Disease-free survival
LRR: Local recurrence rate
NAC: Neoadjuvant chemotherapy
