# Supplementary figures and images for: Prognosis and local treatment strategies of breast cancer patients with different numbers of micrometastatic lymph nodes
Source: World J Surg Oncol. 2023 Jul 10;21:202. doi: 10.1186/s12957-023-03082-x (PMC10332040; doi:10.1186/s12957-023-03082-x)

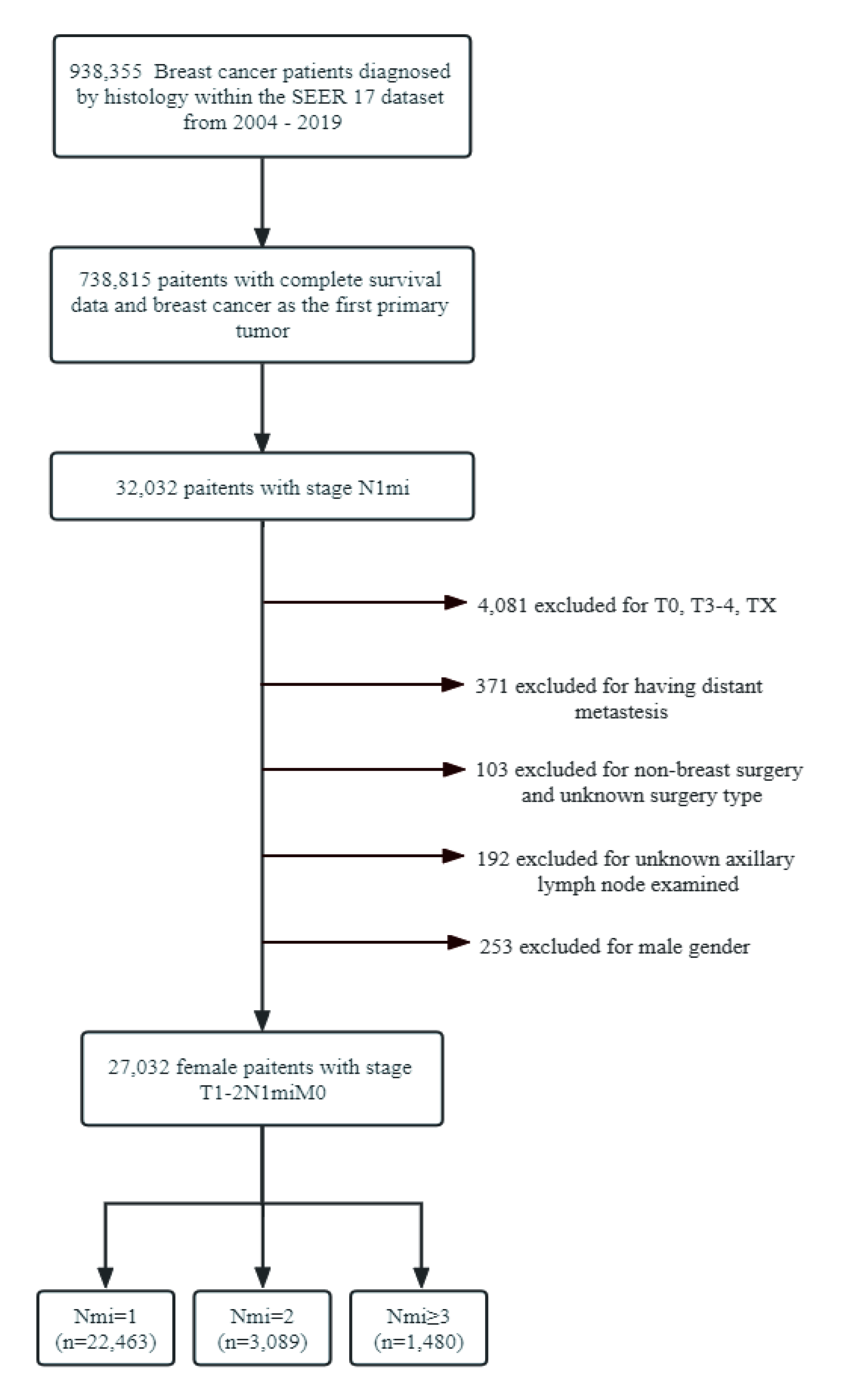

Supplement: Supplementary file 1 — Additional file 1: Supplemental Figure S1. Flow diagram of identifying eligible patients with T1-2N1miM0 breast cancer. [file 12957_2023_3082_MOESM1_ESM.tif]

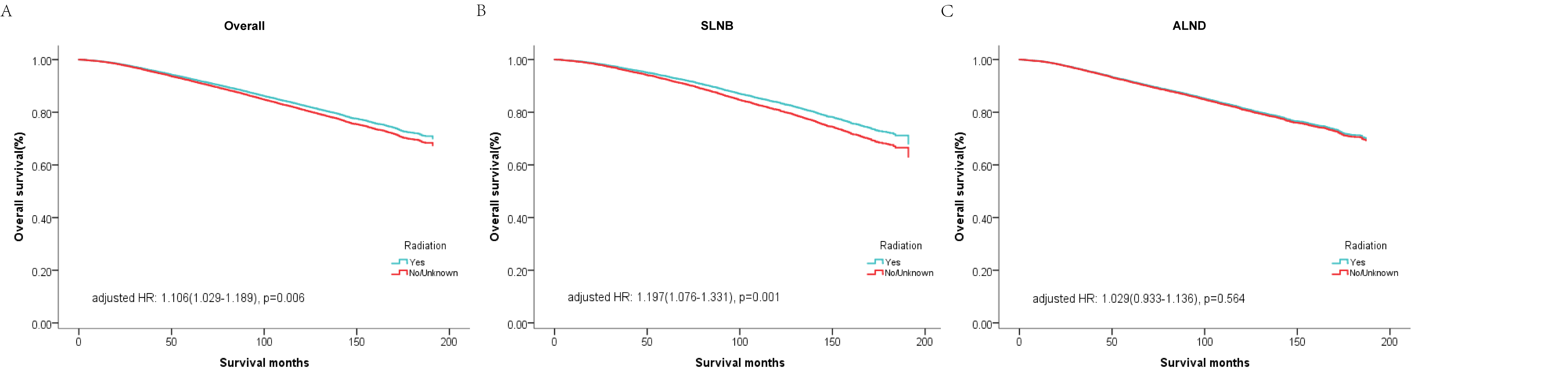

Supplement: Supplementary file 2 — Additional file 2: Supplemental Figure S2. The survival curves of receiving radiotherapy or not, adjusted by other prognostic factors. [file 12957_2023_3082_MOESM2_ESM.tif]
