# Supplementary material for: Prognosis and local treatment strategies of breast cancer patients with different numbers of micrometastatic lymph nodes
Source: World J Surg Oncol. 2023 Jul 10;21:202. doi: 10.1186/s12957-023-03082-x (PMC10332040; doi:10.1186/s12957-023-03082-x)
Supplement: Supplementary file 3 — Additional file 3: Supplemental Table S1. Univariate and multivariate analyses of overall survival (OS) in T1-2N1miM0 patients. [file 12957_2023_3082_MOESM3_ESM.docx]

**Supplemental Table 1.** Univariate and multivariate analyses of overall survival (OS) in T1-2N1miM0 patients

| **Variables** | **Univariate regression** | | **Multivariate regression** | |
| --- | --- | --- | --- | --- |
|  | **HR (95%CI)** | ***P* value** | **HR (95%CI)** | ***P* value** |
| **Age，years** |  | **<0.001** |  | **<0.001** |
| ＜40 | 1.000 |  | 1.000 |  |
| 40-59 | 0.794(0.690-0.915) | **0.001** | 0.896(0.778-1.033) | **0.131** |
| ≥60 | 2.371(2.071-2.714) | **<0.001** | 2.333(2.030-2.681) | **<0.001** |
| **Race** |  | **<0.001** |  | **<0.001** |
| White | 1.000 |  | 1.000 |  |
| Black | 1.427(1.310-1.556) | **<0.001** | 1.252(1.146-1.367) | **<0.001** |
| Other ^a^ | 0.713(0.634-0.802) | **<0.001** | 0.767(0.682-0.863) | **<0.001** |
| **Marital** |  | **<0.001** |  | **<0.001** |
| Married | 1.000 |  | 1.000 |  |
| Single ^b^ | 1.867(1.759-1.982) | **<0.001** | 1.490(1.402-1.584) | **<0.001** |
| Unknown | 1.305(1.107-1.539) | **0.002** | 1.109(0.940-1.309) | **0.022** |
| **Histological types** |  | **<0.001** |  | **<0.001** |
| IDC | 1.000 |  | 1.000 |  |
| ILC | 1.060(0.952-1.181) | 0.289 | 0.991(0.886-1.109) | 0.878 |
| Other | 0.845(0.776-0.920) | **<0.001** | 0.822(0.764-0.907) | **<0.001** |
| **Grade** |  | **<0.001** |  | **<0.001** |
| I | 1.000 |  | 1.000 |  |
| II | 1.315(1.203-1.437) | **<0.001** | 1.247(1.140-1.365) | **<0.001** |
| III | 1.806(1.650-1.976) | **<0.001** | 1.628(1.474-1.798) | **<0.001** |
| Unknown | 1.253(1.035-1.517) | **0.021** | 1.072(0.883-1.301) | **0.483** |
| **T Stage** |  | **<0.001** |  | **<0.001** |
| T1 | 1.000 |  | 1.000 |  |
| T2 | 1.664(1.570-1.765) | **<0.001** | 1.540(1.448-1.638) | **<0.001** |
| **Nodal Status** |  | **<0.001** |  | **<0.001** |
| **N1mi=1** | **1.000** |  | **1.000** |  |
| **N1mi=2** | **1.122(1.027-1.223)** | **0.011** | **1.145(1.047-1.251)** | **0.003** |
| **N1mi≥3** | **1.746(1.583-1.926)** | **<0.001** | **1.697(1.530-1.882)** | **<0.001** |
| **Type of Surgery** |  | **<0.001** |  | **<0.001** |
| BCS | 1.000 |  | 1.000 |  |
| Mastectomy | 1.264(1.192-1.340) | **<0.001** | 1.123(1.045-1.208) | **0.002** |
| **Type of Axillary Surgery** |  | **0.359** |  | **0.033** |
| SLNB | 1.000 |  | 1.000 |  |
| ALND | **1.028(0.969-1.091)** | **0.359** | **0.932(0.874-0.994)** | **0.033** |
| **Radiation** |  | **<0.001** |  | **0.006** |
| Yes | 1.000 |  | **1.000** |  |
| No/Refused | **1.292(1.218-1.371)** | **<0.001** | **1.107(1.030-1.190)** | **0.006** |
| **Chemotherapy** |  | **<0.001** |  | **<0.001** |
| Yes | 1.000 |  | 1.000 |  |
| No/Unknown | 1.763(1.662-1.871) | **<0.001** | 1.620(1.515-1.732) | **<0.001** |
| **ER Status** |  | **<0.001** |  | **<0.001** |
| Positive | 1.000 |  | 1.000 |  |
| Negative | 1.740(1.615-1.875) | **<0.001** | 1.301(1.171-1.445) | **<0.001** |
| Borderline | 1.507(1.291-1.759) | **<0.001** | 1.199(0.916-1.569) | **0.186** |
| **PR Status** |  | **<0.001** |  | **<0.001** |
| Positive | 1.000 |  | 1.000 |  |
| Negative | 1.754(1.645-1.869) | **<0.001** | 1.375(1.259-1.502) | **<0.001** |
| Borderline | 1.503(1.311-1.722) | **<0.001** | 1.150(0.908-1.456) | **0.247** |
| **HER2 Status** |  | **0.012** |  | **0.002** |
| Positive | 1.000 |  | 1.000 |  |
| Negative | 1.258(1.069-1.480) | **0.006** | 1.329(1.126-1.568) | **0.001** |
| Borderline | 1.460(1.118-1.906) | **0.005** | 1.287(0.982-1.688) | **0.068** |
| Not 2010+ | 1.281(1.092-1.504) | **0.002** | 1.379(1.173-1.622) | **<0.001** |

*HR* hazard ratio, *CI* confidence interval
