# Supplementary material for: Prognosis and local treatment strategies of breast cancer patients with different numbers of micrometastatic lymph nodes
Source: World J Surg Oncol. 2023 Jul 10;21:202. doi: 10.1186/s12957-023-03082-x (PMC10332040; doi:10.1186/s12957-023-03082-x)
Supplement: Supplementary file 4 — Additional file 4: Supplemental Table S2. Baseline characteristics of before and after matching in patients, stratified by type of axillary surgery. [file 12957_2023_3082_MOESM4_ESM.docx]

**Supplemental Table 2.** Baseline characteristics of before and after matching in patients**,** stratified by type of axillary surgery

| **Characteristics** |  | **Before Matching** | | | **After Matching** | | |
| --- | --- | --- | --- | --- | --- | --- | --- |
|  |  | **SLNB** | **ALND** | ***P* value** | **SLNB** | **ALND** | ***P* value** |
| n |  | 15622 | 11410 |  | 8334 | 8334 |  |
| **Age，years** | ＜40 | 886 (5.7) | 952 ( 8.3) | <0.001 | 633 ( 7.6) | 652 ( 7.8) | 0.515 |
|  | 40-59 | 7197 ( 46.1) | 5944 ( 52.1) |  | 4194 ( 50.3) | 4246 ( 50.9) |  |
|  | ≥60 | 7539 ( 48.3) | 4514 ( 39.6) |  | 3507 ( 42.1) | 3436 ( 41.2) |  |
| **Race** | White | 12602 ( 80.7) | 8864 ( 77.7) | <0.001 | 6598 ( 79.2) | 6578 ( 78.9) | 0.096 |
|  | Black | 1380 ( 8.8) | 1413 ( 12.4) |  | 897 ( 10.8) | 844 ( 10.1) |  |
|  | Other ^a^ | 1640 ( 10.5) | 1133 ( 9.9) |  | 839 ( 10.1) | 912 ( 10.9) |  |
| **Marital** | Married | 9307 ( 59.6) | 6823 ( 59.8) | 0.356 | 4890 ( 58.7) | 5015 ( 60.2) | 0.091 |
|  | Single ^b^ | 5668 ( 36.3) | 4154 ( 36.4) |  | 3141 ( 37.7) | 3005 ( 36.1) |  |
|  | Unknown | 647 ( 4.1) | 433 ( 3.8) |  | 303 ( 3.6) | 314 ( 3.8) |  |
| **Histological types** | IDC | 12158 ( 77.8) | 8912 ( 78.1) | <0.001 | 6494 ( 77.9) | 6512 ( 78.1) | 0.935 |
|  | ILC | 1323 ( 8.5) | 800 ( 7.0) |  | 609 ( 7.3) | 599 ( 7.2) |  |
|  | Other | 2141 ( 13.7) | 1698 ( 14.9) |  | 1231 ( 14.8) | 1223 ( 14.7) |  |
| **Grade** | I | 3411 ( 21.8) | 1841 ( 16.1) | <0.001 | 1468 ( 17.6) | 1525 ( 18.3) | 0.698 |
|  | II | 7781 ( 49.8) | 5169 ( 45.3) |  | 3927 ( 47.1) | 3886 ( 46.6) |  |
|  | III | 4100 ( 26.2) | 4067 ( 35.6) |  | 2722 ( 32.7) | 2713 ( 32.6) |  |
|  | Unknown | 330 ( 2.1) | 333 ( 2.9) |  | 217 ( 2.6) | 210 ( 2.5) |  |
| **T Stage** | T1 | 9686 ( 62.0) | 6300 ( 55.2) | <0.001 | 4883 ( 58.6) | 4883 ( 58.6) | 1.000 |
|  | T2 | 5936 ( 38.0) | 5110 ( 44.8) |  | 3451 ( 41.4) | 3451 ( 41.4) |  |
| **Nodal Status** | **N1mi=1** | 14293 ( 91.5) | 8170 ( 71.6) | <0.001 | 7208 ( 86.5) | 7114 ( 85.4) | 0.091 |
|  | **N1mi=2** | 1176 ( 7.5) | 1913 ( 16.8) |  | 973 ( 11.7) | 1043 ( 12.5) |  |
|  | **N1mi≥3** | 153 ( 1.0) | 1327 ( 11.6) |  | 153 ( 1.8) | 177 ( 2.1) |  |
| **Type of Surgery** | BCS | 9907 ( 63.4) | 4919 ( 43.1) | <0.001 | 4147 ( 49.8) | 4190 ( 50.3) | 0.515 |
|  | Mastectomy | 5715 ( 36.6) | 6491 ( 56.9) |  | 4187 ( 50.2) | 4144 ( 49.7) |  |
| **Radiation** | Yes | 9354 ( 59.9) | 5014 ( 43.9) | <0.001 | 3934 ( 47.2) | 4003 ( 48.0) | 0.292 |
|  | No/Refused | 6268 ( 40.1) | 6396 ( 56.1) |  | 4400 ( 52.8) | 4331 ( 52.0) |  |
| **Chemotherapy** | Yes | 6894 ( 44.1) | 7145 ( 62.6) | <0.001 | 4783 ( 57.4) | 4785 ( 57.4) | 0.988 |
|  | No/Unknown | 8728 ( 55.9) | 4265 ( 37.4) |  | 3551 ( 42.6) | 3549 ( 42.6) |  |
| **ER Status** | Positive | 13872 ( 88.8) | 9336 ( 81.8) | <0.001 | 7038 ( 84.4) | 7046 ( 84.5) | 0.344 |
|  | Negative | 1499 ( 9.6) | 1772 ( 15.5) |  | 1097 ( 13.2) | 1116 ( 13.4) |  |
|  | Borderline | 251 ( 1.6) | 302 ( 2.6) |  | 199 ( 2.4) | 172 ( 2.1) |  |
| **PR Status** | Positive | 12472 ( 79.8) | 8159 ( 71.5) | <0.001 | 6188 ( 74.3) | 6198 ( 74.4) | 0.615 |
|  | Negative | 2815 ( 18.0) | 2834 ( 24.8) |  | 1874 ( 22.5) | 1886 ( 22.6) |  |
|  | Borderline | 335 ( 2.1) | 417 ( 3.7) |  | 272 ( 3.3) | 250 ( 3.0) |  |
| **HER2 Status** | Positive | 1248 ( 8.0) | 800 ( 7.0) | <0.001 | 712 ( 8.5) | 654 ( 7.8) | 0.015 |
|  | Negative | 9860 ( 63.1) | 4097 ( 35.9) |  | 3695 ( 44.3) | 3574 ( 42.9) |  |
|  | Borderline | 363 ( 2.3) | 237 ( 2.1) |  | 211 ( 2.5) | 192 ( 2.3) |  |
|  | Not 2010+ | 4151 ( 26.6) | 6276 ( 55.0) |  | 3716 ( 44.6) | 3914 ( 47.0) |  |
