# Supplementary material for: Prognosis and local treatment strategies of breast cancer patients with different numbers of micrometastatic lymph nodes
Source: World J Surg Oncol. 2023 Jul 10;21:202. doi: 10.1186/s12957-023-03082-x (PMC10332040; doi:10.1186/s12957-023-03082-x)
Supplement: Supplementary file 5 — Additional file 5: Supplemental Table S3. The number of patients with T1-2N1miM0 breast cancer undergoing different types of axillary surgery between 2004 and 2019. [file 12957_2023_3082_MOESM5_ESM.docx]

**Supplemental Table 3.** The number of patients with T1-2N1miM0 breast cancer undergoing different types of axillary surgery between 2004 and 2019.

|  | Total cohort | | | | Nmi=1 | | | | Nmi=2 | | | |
| --- | --- | --- | --- | --- | --- | --- | --- | --- | --- | --- | --- | --- |
| Year of diagnosis | Total | SLNB | ALND | P % ^a^ | Total | SLNB | ALND | P % | Total | SLNB | ALND | P % |
| Total | 27031 | 15622 | 11410 | 57.79% | 22463 | 14293 | 8170 | 63.63% | 3089 | 1176 | 1913 | 38.07% |
| 2004 | 1616 | 656 | 960 | 40.59% | 1270 | 592 | 678 | 46.61% | 215 | 53 | 162 | 24.65% |
| 2005 | 1774 | 687 | 1087 | 38.73% | 1356 | 618 | 738 | 45.58% | 257 | 63 | 194 | 24.51% |
| 2006 | 1716 | 678 | 1038 | 39.51% | 1328 | 613 | 715 | 46.16% | 233 | 53 | 180 | 22.75% |
| 2007 | 1770 | 712 | 1058 | 40.23% | 1407 | 644 | 763 | 45.77% | 226 | 57 | 169 | 25.22% |
| 2008 | 1785 | 716 | 1069 | 40.11% | 1431 | 646 | 785 | 45.14% | 242 | 65 | 177 | 26.86% |
| 2009 | 1765 | 702 | 1063 | 39.77% | 1449 | 648 | 801 | 44.72% | 200 | 43 | 157 | 21.50% |
| 2010 | 1725 | 787 | 938 | 45.62% | 1385 | 718 | 667 | 51.84% | 228 | 61 | 167 | 26.75% |
| 2011 | 1857 | 1123 | 734 | 60.47% | 1507 | 1023 | 484 | 67.88% | 232 | 94 | 138 | 40.52% |
| 2012 | 1787 | 1197 | 590 | 66.98% | 1492 | 1088 | 404 | 72.92% | 197 | 94 | 103 | 47.72% |
| 2013 | 1674 | 1111 | 563 | 66.37% | 1399 | 1014 | 385 | 72.48% | 182 | 87 | 95 | 47.80% |
| 2014 | 1700 | 1213 | 487 | 71.35% | 1481 | 1117 | 364 | 75.42% | 159 | 81 | 78 | 50.94% |
| 2015 | 1714 | 1284 | 430 | 74.91% | 1472 | 1179 | 293 | 80.10% | 185 | 96 | 89 | 51.89% |
| 2016 | 1509 | 1131 | 378 | 74.95% | 1354 | 1060 | 294 | 78.29% | 132 | 63 | 69 | 47.73% |
| 2017 | 1530 | 1184 | 346 | 77.39% | 1359 | 1080 | 279 | 79.47% | 144 | 100 | 44 | 69.44% |
| 2018 | 1567 | 1209 | 358 | 77.15% | 1396 | 1122 | 274 | 80.37% | 128 | 77 | 51 | 60.16% |
| 2019 | 1543 | 1232 | 311 | 79.84% | 1377 | 1131 | 246 | 82.14% | 129 | 89 | 40 | 68.99% |

*SLNB* Sentinel lymph node biopsy; *ALND* axillary lymph node dissection

^a^ P % represents the proportion of undergoing SLNB
