# Supplementary material for: Prognosis and local treatment strategies of breast cancer patients with different numbers of micrometastatic lymph nodes
Source: World J Surg Oncol. 2023 Jul 10;21:202. doi: 10.1186/s12957-023-03082-x (PMC10332040; doi:10.1186/s12957-023-03082-x)
Supplement: Supplementary file 6 — Additional file 6: Supplemental Table S4. Baseline characteristics of before and after matching in patients, stratified by receive radiation or not. [file 12957_2023_3082_MOESM6_ESM.docx]

**Supplemental Table 4.** Baseline characteristics of before and after matching in patients**,** stratified by receive radiation or not

| **Characteristics** |  | **SLNB** | **ALND** | ***P* value** |
| --- | --- | --- | --- | --- |
| n |  | 14368 | 12664 |  |
| **Age，years** | <40 | 938 ( 6.5) | 900 ( 7.1) | 0.003 |
|  | 40-59 | 6893 ( 48.0) | 6248 ( 49.3) |  |
|  | ≥60 | 6537 ( 45.5) | 5516 ( 43.6) |  |
| **Race** | White | 11492 ( 80.0) | 9974 ( 78.8) | 0.036 |
|  | Black | 1430 ( 10.0) | 1363 ( 10.8) |  |
|  | Other | 1446 ( 10.1) | 1327 ( 10.5) |  |
| **Marital** | Married | 8798 ( 61.2) | 7332 ( 57.9) | <0.001 |
|  | Single | 5094 ( 35.5) | 4728 ( 37.3) |  |
|  | Unknown | 476 ( 3.3) | 604 ( 4.8) |  |
| **Histological types** | IDC | 11451 ( 79.7) | 9619 ( 76.0) | <0.001 |
|  | ILC | 1066 ( 7.4) | 1057 ( 8.3) |  |
|  | Other | 1851 ( 12.9) | 1988 ( 15.7) |  |
| **Grade** | I | 2992 ( 20.8) | 2260 ( 17.8) | <0.001 |
|  | II | 6913 ( 48.1) | 6037 ( 47.7) |  |
|  | III | 4183 ( 29.1) | 3984 ( 31.5) |  |
|  | Unknown | 280 ( 1.9) | 383 ( 3.0) |  |
| **T Stage** | T1 | 8832 ( 61.5) | 7154 ( 56.5) | <0.001 |
|  | T2 | 5536 ( 38.5) | 5510 ( 43.5) |  |
| **Nodal Status** | N1mi=1 | 11995 ( 83.5) | 10468 ( 82.7) | 0.181 |
|  | N1mi=2 | 1598 ( 11.1) | 1491 ( 11.8) |  |
|  | N1mi≥3 | 775 ( 5.4) | 705 ( 5.6) |  |
| **Type of Surgery** | BCS | 11525 ( 80.2) | 3301 ( 26.1) | <0.001 |
|  | Mastectomy | 2843 ( 19.8) | 9363 ( 73.9) |  |
| **Type of Axillary Surgery** | SLNB | 9354 ( 65.1) | 6268 ( 49.5) | <0.001 |
|  | ALND | 5014 ( 34.9) | 6396 ( 50.5) |  |
| **Chemotherapy** | Yes | 7874 ( 54.8) | 6165 ( 48.7) | <0.001 |
|  | No/Unknown | 6494 ( 45.2) | 6499 ( 51.3) |  |
| **ER Status** | Positive | 12616 ( 87.8) | 10592 ( 83.6) | <0.001 |
|  | Negative | 1577 ( 11.0) | 1694 ( 13.4) |  |
|  | Borderline | 175 ( 1.2) | 378 ( 3.0) |  |
| **PR Status** | Positive | 11343 ( 78.9) | 9288 ( 73.3) | <0.001 |
|  | Negative | 2776 ( 19.3) | 2873 ( 22.7) |  |
|  | Borderline | 249 ( 1.7) | 503 ( 4.0) |  |
| **HER2 Status** | Positive | 1074 ( 7.5) | 974 ( 7.7) | <0.001 |
|  | Negative | 8083 ( 56.3) | 5874 ( 46.4) |  |
|  | Borderline | 229 ( 1.6) | 371 ( 2.9) |  |
|  | Not 2010+ | 4982 ( 34.7) | 5445 ( 43.0) |  |
